# Functional Immunomics of the Squash Bug, *Anasa tristis* (De Geer) (Heteroptera: Coreidae)

**DOI:** 10.3390/insects4040712

**Published:** 2013-11-26

**Authors:** Kent S. Shelby

**Affiliations:** Biological Control of Insects Research Laboratory, United States Department of Agriculture, Agricultural Research Service, 1503 S. Providence Road, Columbia, MO 65203, USA; E-Mail: kent.shelby@ars.usda.gov; Tel.: +1-573-876-8308; Fax: +1-573-875-5364

**Keywords:** squash bug, cucurbits, expression profiling, RNA-seq, entomopathogen response, PAMPs, PRRs, anacin

## Abstract

The Squash bug, *Anasa tristis* (De Geer), is a major piercing/sucking pest of cucurbits, causing extensive damage to plants and fruits, and transmitting phytopathogens. No genomic resources to facilitate field and laboratory studies of this pest were available; therefore the first *de novo* exome for this destructive pest was assembled. RNA was extracted from insects challenged with bacterial and fungal immunoelicitors, insects fed on different cucurbit species, and insects from all life stages from egg to adult. All treatments and replicates were separately barcoded for subsequent analyses, then pooled for sequencing in a single lane using the Illumina HiSeq2000 platform. Over 211 million 100-base tags generated in this manner were trimmed, filtered, and cleaned, then assembled into a *de novo* reference transcriptome using the Broad Institute Trinity assembly algorithm. The assembly was annotated using NCBIx NR, BLAST2GO, KEGG and other databases. Of the >130,000 total assemblies 37,327 were annotated identifying the sequences of candidate gene silencing targets from immune, endocrine, reproductive, cuticle, and other physiological systems. Expression profiling of the adult immune response was accomplished by aligning the 100-base tags from each biological replicate from each treatment and controls to the annotated reference assembly of the *A. tristis* transcriptome.

## 1. Introduction

The squash bug, *Anasa tristis* (De Geer), is a major pest of squash, pumpkin, watermelon, cucumber and cantaloupe production, causing substantial economic losses throughout its range [1]. Squash bug feeding causes extensive damage to stems resulting in wilting, fruit discoloration and pre/postharvest spoilage. Possibly more important, *A. tristis* vectors the causal bacterial agent of Cucurbit yellow vine disease (CYVD), *Serratia marcescens* [2]. This serious disease was first recorded in Texas and Oklahoma in 1988 and is now rapidly spreading through the West and Midwest. The epidemiology of CYVD ranges from little impact in some years to regional crop failure in others. Natural enemies of the squash bug include the scelionid wasp egg parasitoid *Gryon pennsylvanicum* (Ashmead), the tachinid parasitoid of adult squash bugs, *Tricopoda pennipes*) [3,4,5], and several predatory arthropods [6]*. A. tristis* adults enter diapause in the Fall, and in the Midwest *A. tristis* adults terminate diapause in May then proceed to feed on host plants [7,8]. *A. tristis* are univoltine exhibiting one generation each summer season beginning and ending with overwintering adults [8]. There are few effective biological agents or cultural practices for controlling this highly destructive pest aside from insecticides, thus development of more effective control measures are much needed. In their natural environments insects are subjected to a high incidence of opportunistic microbial infection [9] and parasitization [10]; thus immunoevasion and immunosuppression of the insect host is a strategy widely in use by parasitoids, nematodes, trypanosomes, bacteria and viruses [11]. Experimental immunosuppression of insects *via* malnutrition [10], or by dietary deficiencies of selenium [12,13,14,15] or of ascorbic acid [16,17,18] can enhance entomopathogen virulence. Immunosuppression of host larvae by injection of double stranded RNA directed against immune system components also increases susceptibility to microbial entomopathogens [19,20,21,22,23]. Field application of RNAi targeted specifically against insect pests is a promising new control approach [24,25,26]. Demonstration of field efficacy on a commercial scale has already been demonstrated by *in situ* oral vaccination of honey bees against the Israeli Acute Paralysis Virus by inclusion of RNAi against the virus in feeding solutions [27,28]. However, delivery of a sufficient quantity of RNAi to adversely impact pest life processes is far more challenging in the case of piercing/sucking insect pests, requiring plant-based expression of RNAi [29,30,31,32], although soaking or spraying with RNAi may become feasible in the future for some crops [26].

Deployment of RNAi against pest insects requires the discovery and generation of species specific gene silencing targets which disrupt tissues of targeted pests [31,32,33], or block central metabolic pathways such as arginine kinase [34] or mitochondrial Rieske iron-sulfur protein [35]. Immunosuppression as a biological control strategy [36] would first require discovery of viable, easily accessible gene silencing targets from among the squash bug immune system. The objective of this study therefore was to identify candidate immunosuppressive gene silencing targets suitable for control of *A. tristis*. The sequences of many inducible and constitutively expressed immune system components orthologous to those of other insect species were identified, and in addition a transcriptome was compiled which contains the vast bulk of *A. tristis* unigenes enabling a wide range of studies using this pest insect.

## 2. Experiment

### 2.1. Insects, Infections and RNA Isolation

Colonies were founded by collection of adult squash bugs, *Anasa tristis* (De Geer) (NCBI Taxonomy ID: 236421), from squash and zucchini plants at the USDA Agricultural Research Service Biological Control of Insects Research Laboratory (38.934159°N, −92.336876°W; Boone Co., Missouri, MO, USA) in June-August, 2010–2011. Colonies were maintained on three week old summer squash plants, a non-disease resistant hybrid yellow crookneck cultivar (*Cucurbita pepo* cv. Dixie; Willhite Seed Co., Poolville, TX, USA) and fruit under standard conditions of 14 h:10 h (L:D) photoperiod, 55% RH, 28 °C with fluorescent, incandescent and LED lighting. Summer squash, butternut squash and zucchini fruit purchased from local organic markets was provided in addition to squash plants. To activate the antibacterial immune response, adults and 5th instar nymphs were subjected to septic puncture with a tungsten needle dipped into a 1 μg/mL suspension of lipopolysaccharide and peptidoglycan (Sigma Chem. Co., St. Louis, MO, USA) in PBS. The antifungal response was activated by puncture with a tungsten needle dipped into a 1 μg/mL suspension of β-glucan, curdlan and laminarin (Sigma Chem. Co., St. Louis, MO, USA) in PBS [37]. Mock controls received a sterile puncture. At each time point 0–18 hours post-inoculation (h.p.i.) animals were flash frozen on dry ice and stored at −85 °C for later use. Three or more complete, separate biological replicates were collected for each treatment time series. Total RNA was extracted by homogenization with a Tissue Tearor (BioSpec Products, Inc., Bartlesville, OK, USA) in TriReagent (Sigma Chem. Co., St. Louis, MO, USA). Total RNA was further cleaned with RNeasy™ kits (Qiagen, Valencia, CA, USA). At least three full biological replicates were collected for each time point. 

### 2.2. Illumina Sequence Generation and Assembly Procedures

RNA was extracted from insects challenged with bacterial and fungal immunoelicitors, insects fed on different cucurbit species, and insects from all life stages from egg to adult. All treatments and replicates were separately barcoded for subsequent analyses, then pooled for sequencing in a single lane using the Illumina HiSeq2000 platform. RNA pools were submitted to University of Missouri Bond Life Sciences Center DNA Core for Illumina GAII sequencing Libraries were constructed following the manufacturer’s protocol with reagents supplied in Illumina’s TruSeq RNA sample preparation kit (#RS-930-2001, Illumina Inc., San Diego, CA, USA). Libraries were constructed according to the standard Illumina RNA-seq protocol (Part# 1004898 Rev. A, rev Sept 08; Illumina Inc., San Diego, CA, USA) from the pooled PCR products except for the fragmentation step as detailed [37,38]. 

Over 211 million ~100-base long Illumina single end reads were first cleaned of low quality bases at the 3’ end, then for low quality overall using the FASTX Toolkit (Cold Spring Harbor Laboratory, Cold Spring Harbor, NY, USA). The remaining reads were screened for homology to contaminants (predominantly rRNA) leaving 115,101,048 reads. After trimming, filtering and cleaning the remaining reads were assembled into a *de novo* reference transcriptome using a local installation of the Broad Institute Trinity assembly algorithm [39]. The assembly was annotated using NCBIx NR, NCBI GVBRL, BLAST2GO [40,41], KEGG and other databases. Of the >130,000 total assemblies 37,327 were annotated including “hypothetical” or “predicted” proteins. Contigs and singletons <80 bases were not analyzed. Annotation of KEGG orthologies (KOs) and metabolic pathway mapping was accomplished using the utilities provided by Kyoto Encyclopedia of Genes and Genomes. All sequence data discussed in this manuscript have been deposited in NCBI GenBank. 

### 2.3. Identification of Differentially Regulated Genes

Expression profiling of *A. tristis* immune system responses were analyzed by normalizing the number of times sequence tags aligning with an mRNA was detected in controls [38]. To accomplish expression profiling the 100-base tags from each treatment, controls and replicates were independently aligned to the annotated assembly of the *A. tristis* reference transcriptome. The final assembly output was piped into a tab-delimited file that was imported into an Excel spreadsheet, which includes for each assembled contig the number of reads and the list of unique names for each read, to facilitate counting the contribution of different libraries for the final assembly. Genes those were upregulated or down regulated by a factor of three and that had an e-value less than 0.0001 are shown in Tables.

## 3. Results and Discussion

### 3.1. Anasa Tristis RNA-Seq

**Figure 1:**
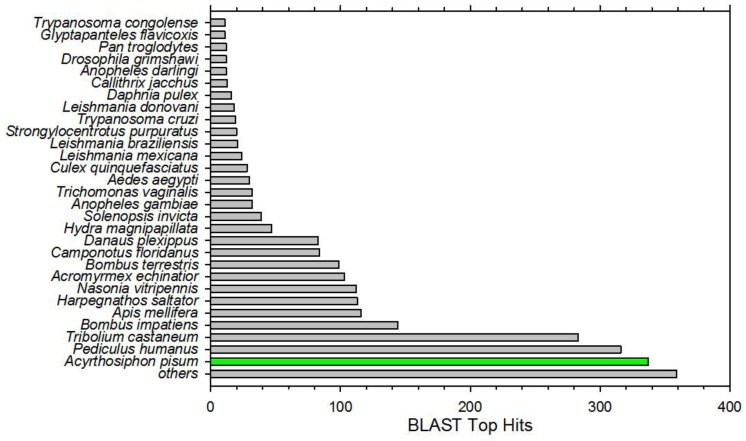
*Anasa tristis* transcriptome assembly Top BLAST hits across insects and other invertebrate species. Highest number of hits was against the insect species most closely related to *A. tristis*, and which has a sequenced genome, *Acyrthosiphon pisum* (green).

A total of 211,532,043 reads were acquired from the 11 barcoded input cDNA libraries. After quality trimming and filtering with FASTx 54.4% of the initial reads, 115,101,048 reads, remained for the assembly (Table 1). These were assembled using the program Trinity [39]. Over 211 million 100-base tags generated in this manner were trimmed, filtered, and cleaned, then assembled into a *de novo* reference transcriptome using the Broad Institute Trinity assembly algorithm. Annotation of this assembly [40] resulted in the identification of 37,327 unigenes (Table 1) of which the majority were genuine orthologs of Arthropoda (Figure 1). The read length of those over 300 bp averaged 1588 bp (range 301 bp to 16,368 bp) (Table 1). In comparison to other insect species this is likely an overestimate of the actual number of genes present in *A. tristis*. However a better estimate will not be available without alignment to a completed *A. tristis* reference genome. Genome size estimates of squash bug adults from this colony were estimated to be 1726 ± 29.2 Mb (♂) and 1782 ± 14.6 Mb (♀) [42].

**Table 1:** Assembly and Annotation Summary of Illumina sequence Tags Derived from Immune-Stimulated Adult and Nymphal *Anasa tristis*.

Parameter	Output
Total Sequences	211, 532, 043
Input Sequences	115, 101, 048 (54.4%)
Contigs ^a^	130,000
Contigs > 300 bp	96,480
Annotated ^b^	
Contigs >300 bp	37,327
	(Ave = 1,588 bp; Range 301 to 16,368)
Contigs > 5 kb	858
Contigs > 4 kb < 5 kb	1,068
Contigs > 3 kb < 4 kb	2,450
Contigs > 2 kb < 3 kb	5,601
Contigs > 1 kb < 2 kb	12,216
Contigs > 301 bp < 1 kb	15,135

^a^ Trinity Assembly [40].^b^ Significant BLAST score (e > 10^−6^) using BLAST2GO [41].

**Figure 2:**
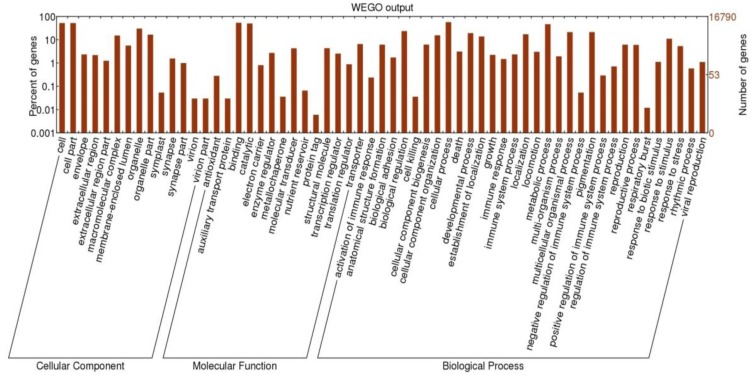
**G**enome ontology analysis of the *Anasa tristis* transcriptome assembly. BGI WEGO plot of Cellular Component, Molecular Function, and Biological Process GOs are shown.

Genome Ontology annotation of these contigs demonstrated that the RNA pools constituting the assembly resulted in successful sampling of the major classes of cellular component, molecular function and biological processes. A complete set of cytoplasmic and mitochondrial tRNAs was assembled, and the majority of tRNA-acyltransferases. GO annotation revealed substantial acquisition of transcripts involved in reproduction and reproductive processes (2,043), central metabolism (7,818), cell and organellar proliferation, division and movement (9,580), physiological processes (3,571), and arthropod specific functions such as molting and pupation (911). KEGG maps generated using BLAST2GO demonstrated that major metabolic pathways were reasonably complete, constituting a majority of enzymes required. The experiments were specifically designed to sample *A. tristis* immune system processes and the annotation resulted in the identification of 394 GOs from this category, and in addition 169 immune system regulatory GOs, 76 coagulation, and two respiratory burst GOs were noted (Figure 2).

Contamination with squash and microbial sequences was expected because whole adults and nymphs actively feeding upon live cucurbits were used to generate RNA pools. Also an effort was made to filter and remove contaminating sequences before assembly of the transcriptome. Despite this only a single contaminating sequence annotating as a putative annexin ortholog of *Cucumis melo* was identified. This may be partially explained as consistent with the squash bug piercing/sucking mode of feeding which differs from foliar herbivores that retain large masses of partially digested plant material within their midguts posing a greater risk of contamination with plant sequences.

Trace contamination with *Wolbachia spp*., *Burkholderia spp*., *Pseudomonas spp*., Proteobacteria, phytoplasmodia, mycoplasmodia and other bacterial or fungal species was observed. The sample preparation method would have allowed contamination with midgut floral sequences. Also identified were sequences annotating as *Serratia marcescens*, the squash bug vectored bacterial agent of Cucurbit yellow vine disease [2,43]. Phloem feeding insects are famously dependent upon obligate symbiotic bacteria to compensate for nutritional deficiencies inherent to this diet [44,45,46], thus it is highly likely that *A. tristis* also may possess symbiotes as these and other data suggest [47]. A substantial number of sequences had significant matches to kinetoplastids (Figure 1), indicating that the very recently established squash bug colony may harbor these parasites. A metagenomic study of *A. tristis* symbionts and other associated flora would confirm and expand on these tantalizing first hits. Finally, the vast bulk of remaining contigs with significant BLAST scores annotated, as expected, within the Arthropoda (Figure 1), Hemiptera, and in particular were orthologous with the only hemipteran insect having a completed/annotated genome, *Acyrthosiphon pisum*.

An explication of all the putative *A. tristis* unigenes identified in this survey would exceed the ambit of a single manuscript, therefore only key categories of identified immunity unigenes will be highlighted below. 

### 3.2. Immunity Related Transcripts Identified in the *A. Tristis* Transcriptome

Many developmental, rare or environmental transcripts are not adequately sampled by insect transcriptome projects as the treatments needed to induce transcription or particular life stages were not included. Thus the inducible transcripts of the insect immune system are routinely missing from published transcriptomes. In this report a deliberate effort was made to sample transcripts from a variety of environmental insults, developmental stages and immune activation. As no entomopathogens are currently known for *A. tristis*, and because the RNA-seq method is extremely sensitive to contaminating entomopathogen nucleic acid sequences, only purified cell wall components of bacteria and fungi were used to elicit an immune response *in lieu* of live or intact entomopathogens. The immune system of adult squash bugs was activated by septic puncture using either bacterial or fungal elicitors [37]. Control insects were subjected a sterile puncture. Septic puncture of adult squash bugs with bacterial cell wall elicitors resulted in the increased transcription of 3,950 contigs, and decreased levels of 5,242 contigs. Septic puncture with fungal cell wall elicitors resulted in the increased expression of 2,434 transcripts, and decreased transcription of 6,021. Control adults receiving a sterile puncture expressed 1,491 unique transcripts, while 2,088 transcripts were unique to the bacterial elicitation, and 1,941 were unique to the fungal elicitation. An upper bound estimate of the number of genes directly involved in the insect immune response was reported by Brucker *et al*. [48] who identified 489 putative immune system genes by hidden Markov model search of the completed genome of the jewel wasp, *Nasonia vitripennis*.

### 3.3. Pattern Recognition and Signal Transduction

The immune response is triggered by the presence of pathogen associated molecular patterns (PAMPs) such as bacterial cell wall or flagellar components in the presence of host damage associated molecular patterns and chemokines [49]. Pattern recognition receptors located on hemocytes and other tissues bind to the PAMPs activating one or more signal transduction pathways which lead to the synthesis of many antimicrobial enzymes and peptides. Several orthologs of PAMPS and pattern recognition receptors were identified within the *A. tristis* transcriptome. Among these were β-1,3-glucan recognition protein 4a [JQ398676], peptidoglycan recognition protein S2 (JQ398681), C-type lectins, galectins, Dscams, hemolectin, scavenger receptor class B [KF578379], and leucine-rich repeat proteins (Table 2). Elicitation with bacterial or fungal cell wall components via septic puncture led to the upregulation of several PAMPS. Expression of Dscam was upregulated 15-fold by bacterial elicitation, and 10-fold by fungal elicitation. The scavenger receptor ortholog was upregulated 149-fold by bacterial and 124-fold by fungal elicitation of *A. tristis* adults (Table 2).

Three main conserved signal transduction pathways occur in insects: The *toll* pathway responsive to gram-positive bacteria and fungi; the *imd* pathway responsive to gram-negative bacteria; and the JAK/STAT pathway which responds to bacterial or viral infection [50]. Several components of these signal transduction pathways were identified within the assembly. The toll pathway components toll, toll-interacting protein, toll-like receptor 13, dorsal, NF-Қ-B inhibitor alpha, NF-Қ-B inhibitor-interacting Ras-like protein, spaetzle, snake, easter, and relish (Table 2). Among the *imd* pathway components identified were dFADD, JNK-interacting protein 1, JNK-interacting protein 3, (Table 2).

**Table 2:** Expression Profiling of *Anasa tristis* Pattern Recognition and Signal Transduction Unigenes in Response to Bacterial or Fungal Elicitation 18 h post inoculation.

Transcript ID	NR Annotation	MeanControl ^a^	Mean Bacterial ^a^	B/C ^b^	Mean Fungal ^a^	F/C ^b^	NRid	Score	Eval	Best Match
2224c0seq1	15-hydroxyprostaglandin dehydrogenase	325.96	771.19	2.36	508	1.56	ref|XP_001943808.2	204	1E-51	*Acyrthosiphon pisum*
3518c0seq1	β-1,3-glucan recognition protein 4a	306.34	381.82	1.25	363	1.19	gb|ADZ45541.1	247	2E-64	*Hepialus pui*
20931c0seq2	Croquemort	6.74	16.1	2.39	13.17	1.96	gb|EFN76142.1	323	2E-87	*Harpegnathos saltator*
18080c0seq1	C-type Lectin	16.67	5.33	0.32	15	0.9	gb|ABD60991.1	192	6E-49	*Glossina morsitans*
7613c1seq8	Dorsal	29.84	42.47	1.42	33.62	1.13	gb|ABU96698.1	555	1E-157	*Rhodnius prolixus*
17785c0seq3	Dscam	0.96	14.69	15.25	9.83	10.2	ref|XP_003395940.1	1423	0	*Bombus terrestris*
1470c3seq3	FADD	266	313	1.18	134	0.51	ref|NP_001189465.1	61.2	4E-08	*Bombyx mori*
6326c0seq5	Galectin	28.92	82	2.84	39.5	1.37	ref|XP_001656933.1	157	3E-37	*Aedes aegypti*
12793c0seq5	JNK-interacting protein 3	2.07	1.74	0.84	2	0.96	ref|XP_396524.4	428	1E-118	*Apis mellifera*
10217c0seq1	Leucine-rich repeat-containing protein 4B	225	172	0.77	100	0.45	ref|XP_972275.1	526	1E-148	*Tribolium castaneum*
14846c0seq15	MAP kinase-activating death domain	19.3	13.33	0.69	12.28	0.64	gb|EFN64544.1	585	1E-166	*Camponotus floridanus*
2228c0seq2	NF-κ-B inhibitor alpha	132	130	0.99	118	0.9	ref|XP_002427786.1	237	3E-61	*Pediculus humanus*
255c0seq1	PGRP S2-like protein	2462	2134	0.87	2648	1.08	ref|NP_001164436.1	156	2E-37	*Nasonia vitripennis*
109335c0seq1	Phospholipase A2 inhibitor 31 kDa	2	0	0	0.33	0.16	ref|XP003222879.1	87	1E-15	*Anolis carolinensis*
7436c0seq2	Phospholipase A2, iPLA-2	282.73	254	0.89	174	0.61	ref|XP_970721.2	631	1E-179	*Tribolium castaneum*
9865c1seq1	Phospholipase A-2-activating protein	144.90	157	1.08	107	0.74	ref|XP_002423298.1	445	1E-123	*Pediculus humanus*
5036c1seq1	Phospholipase A2	129.37	117	0.90	141	1.09	ref|NP_001191907.1	201	1E-50	*Acyrthosiphon pisum*
8868c0seq1	Prostaglandin E synthase 2	162	209.3	1.28	125	0.77	ref|XP_002432321.1	202	1E-101	*Pediculus humanus*
28963c0seq2	Prostaglandin E2 receptor EP3 subtype	29	0	0	7.3	0.25	ref|XP_001943957.2	237	4E-61	*Acyrthosiphon pisum*
6943c0seq1	Prostaglandin reductase 1	100	216	2.15	116	1.15	ref|XP_001603755.1	373	1E-102	*Nasonia vitripennis*
111273c0seq1	Relish	0.67	0	0	0	0	gb|ACT66912.1	50.8	3E-06	*Apis andreniformis*
20920c0seq4	Scavenger receptor class B	0.09	12.95	149	10.77	124	ref|XP_001947205.2	173	2E-42	*Acyrthosiphon pisum*
16467c0seq1	Easter	73.33	18.67	0.25	54	0.74	gb|EFN62640.1	176	2E-43	*Camponotus floridanus*
15721c0seq1	Snake	45	52.67	1.17	11	0.24	ref|XP_003247331.1	245	1E-63	*Acyrthosiphon pisum*
36985c0seq2	Spaetzle 4	1	4.56	4.56	2.12	2.12	ref|NP_001153592.1	132	5E-30	*Acyrthosiphon pisum*
19735c0seq1	toll	84	84	1	69	0.83	ref|XP_002424097.1	1514	0	*Pediculus humanus*

^a^ Average number of reads/contig (three biological replicates); ^b^ Ratio of reads, Bacterial/Control or Fungal/Control.

Several orthologs of yet another major insect signal transduction pathway, the eicosanoid pathway [51], were recognized within the *A. tristis* transcriptome assembly. Release of arachidonic acid by phospholipase A_2_ allows the C20 fatty acid to enter a series of oxidative reactions yielding second messenger prostaglandins. These prostaglandins bind to receptors that in turn activate hemocytes, among other actions [52]. Within the Heteroptera, the classic model insect *Rhodnius prolixus* has been documented to possess several components of the eicosanoid pathway interacting with trypanosomal infection [53]. Contigs within the *A. tristis* assembly that annotated as orthologs of several enzymes within this pathway were 15-hydroxyprostaglandin dehydrogenase [NAD^+^], peroxidasin-like, phospholipase A2 [JQ398686], Phospholipase A-2-activating protein, prostaglandin E synthase, prostaglandin reductase, prostaglandin E2 receptor (Table 2). The transcript levels of these enzymes, however, remain unchanged by bacterial or fungal elicitation of adult squash bugs.

### 3.4. Melanization, Coagulation and Antimicrobial Activities

Upon infection and activation *via* the *toll* and *imd* signal transduction pathways insects produce a suite of antimicrobial peptides, enzymes and enzyme inhibitors which act to limit growth of the pathogen [50]. Analysis of immune-induced transcripts demonstrated that this is also the case with *A. tristis* (Table 3). Several contigs orthologous to antibacterial peptides of other insects induced by infection were identified. These included transcripts orthologous to a *Triatoma brasiliensis* defensin-like peptide. In accordance with accepted nomenclature I propose the name “Anasin” [KF578378] for this defensin-like peptide induced in *Anasa tristis* by bacterial elicitation. A second transcript encoding an antimicrobial peptide orthologous to hemiptericin of *Pyrrhocoris apterus* was identified which was elevated 16.6-fold by bacterial elicitation. I propose that this peptide be named “Anacin”. Additional antimicrobial enzymes and protease inhibitors were noted, including lysozymes and short pacifastin-like inhibitors (Table 3).

Melanization reactions catalysed by phenoloxidase are primary immune responses to microbial incursion and parasitization which shroud invaders with melanin, crosslink proteins and directly generate microbicidal free radicals [54,55,56]. Contigs annotating as the major insect melanization enzyme prophenoloxidase, as well as putative serpins and serine proteases within the prophenoloxidase regulatory cascade [50] were recognized (Table 3). Hemolymph coagulation is a well-known defence pathway [57] requiring the action of phenoloxidase, as well as other components. Several orthologs of putative coagulation pathways were noted in the assembly (Table 3).

Generation of antimicrobial reactive oxygen and nitrogen species by hemocytes and other tissues acts to limit microbial growth [58]. *A. tristis* orthologs of the plasma membrane reactive oxygen generator NADPH oxidase driving the respiratory burst phenomenon [59] were identified (Table 2). Also orthologs of the antimicrobial free nitrogen radical generator nitric oxide synthase [19] significantly upregulated 3.4-fold by bacterial elicitation were identified within the assembly. A dual oxidase ortholog of the *Drosophila* midgut which generates reactive oxygen species also was noted. Enzymes responsible for inactivation of reactive oxygen species also were noted including superoxide dismutase, catalase, peroxidase, glutathione peroxidase, and others. Finally, the orthologs of iron storage and transporting proteins transferrin and ferritin were observed. While transcript levels of the ferritin ortholog were significantly upregulated 5.3-fold by bacterial and 3.7-fold by fungal elicitation the transcript levels of transferrin appeared to be unaffected by these treatments (Table 3).

**Table 3:** Expression profiling of *Anasa tristis* antimicrobial and melanization unigenes in response to bacterial or fungal elicitation 18 h post inoculation.

Transcript	NR Annotation	Mean Contro l ^a^	Mean Bacterial ^a^	B/C ^b^	Mean Fungal ^a^	F/C ^b^	NRid	Score	Eval	Best Match
693c0seq4	Alaserpin-like	196.48	246.79	1.26	159.80	0.81	ref|XP003399187.1	240	3E-62	*Bombus terrestris*
3354c0seq1	Amyloid beta A4 precursor	144.59	304.18	2.10	161.73	1.12	ref|XP001943320.1	406	1E-111	*Acyrthosiphon pisum*
1603c0seq10	CLIP-associating protein	307.15	372.83	1.21	305.06	0.99	ref|XP003246351.1	404	1E-111	*Acyrthosiphon pisum*
83136c0seq1	Coagulation factor IX	1.67	5.33	3.20	2.33	1.40	ref|XP001943624.2	256	2E-67	*Acyrthosiphon pisum*
23755c0seq2	Coagulation factor X	1.49	1.87	1.26	4.02	2.70	gb|EFN83879.1	150	2E-35	*Harpegnathos saltator*
4923c0seq1	Complement C1r-B subcomponent	366.47	492.67	1.34	100.31	0.27	ref|XP001120594.2	261	2E-68	*Apis mellifera*
393c1seq1	Defensin “Anasin”	1014.84	533.22	0.53	1665.55	1.64	sp|P80407.1	85.5	2E-16	*Triatoma brasiliensis*
15509c0seq1	Dual oxidase	82.33	102.67	1.25	45.67	0.55	ref|XP_001951113.2	2294	0	*Acyrthosiphon pisum*
8c0seq1	ferritin	48316	56283	1.16	57436	1.19	gb|ABR27877.1	291	3E-77	*Triatoma infestans*
82370c0seq1	Ferritin, heavy subunit	2.67	0.00	0.00	1.67	0.63	gb|ACO15170.1	130	2E-30	*Caligus clemensi*
20023c0seq1	Ferritin, middle subunit	10.67	56.72	5.32	39.93	3.74	gb|ACO10415.1	201	7E-51	*Caligus rogercresseyi*
344c0seq1	Gelsolin precursor	7089.52	4998.67	0.71	1494.70	0.21	ref|XP001657431.1	743	0	*Aedes aegypti*
33115c0seq1	Hemiptericin “Anacin”	1.33	22.07	16.55	3.00	2.25	gb|ABW16857.1	79.7	8E-17	*Pyrrhocoris apterus*
2804c0seq1	Hemolectin	61.16	38.67	0.63	16	0.26	ref|XP_002430354.1	52	7E-06	*Pediculus humanus*
533c2seq8	Lysozyme	167.25	144.80	0.87	118.97	0.71	gb|ABX11554.1	159	7E-38	*Rhodnius prolixus*
2227c0seq2	Multiple coagulation factor deficiency protein	304.36	245.79	0.81	258.63	0.85	gb|EGI57786.1	157	2E-37	*Acromyrmex echinatior*
17478c0seq1	NADPH oxidase	184.00	130.33	0.71	146.67	0.80	tpd|FAA00348.1	58.2	5E-07	*Apis mellifera*
5121c0seq1	Nitric oxide synthase	342.29	1154.75	3.37	160.71	0.47	sp|Q26240.1	1784	0	*Apis mellifera*
8767c1seq1	Plasma kallikrein	11.66	26.19	2.25	17.23	1.48	gb|EFN62475.1	54.3	7E-07	*Camponotus floridanus*
6324c0seq4	Platelet-activating factor	62.28	75.57	1.21	111.93	1.80	ref|XP002427586.1	311	9E-84	*Pediculus humanus*
4507c0seq2	Proclotting enzyme	219.76	357.26	1.63	177.83	0.81	gb|EFN62552.1	296	5E-79	*Camponotus floridanus*
10065c0seq1	Prophenoloxidase subunit 2	107.67	103.33	0.96	53.33	0.50	ref|XP967179.2	688	0	*Tribolium castaneum*
15584c0seq1	Prophenoloxidase subunit A3	16.00	8.00	0.50	8.00	0.50	ref|NP001011627.1	298	3E-80	*Apis mellifera*
14386c0seq4	Serpin	5.50	0.10	0.02	0.00	0.00	gb|EFA12666.1	112	8E-24	*Tribolium castaneum*
254c0seq1	Transferrin	7350.06	14070.74	1.91	10554.93	1.44	gb|AAD02419.1	939	0	*Riptortus clavatus*

^a^ Average number of reads/contig (three biological replicates); ^b^ Ratio of reads, Bacterial/Control, or Fungal/Control.

### 3.5. RNAi Pathways

Although gene silencing *via* injected or *per os* RNAi has been demonstrated in several Hemipteran species few examples of successful Heteropteran gene silencing are published. Here the *A. tristis* orthologs of proteins required for RNAi are presented. Within the *A. tristis* assembly several orthologs of the miRNA processing pathways were identified (Table 4). An ortholog of the nuclear microprocessor complex subunit, DGCR8 was observed, while cytoplasmic subunits argonaute-1, and -2, aubergine, piwi, dicer-1, dicer-2, and a RISC-loading complex subunit were identified. None of the identified orthologs within these pathways appeared to be differentially regulated by microbial elicitation (Table 4). As there are no known viral entomopathogens of *A. tristis*, no viral elicitation was attempted in this report. It should be noted however that viruses were present within the assembly and thus the insects used for the RNA pools may have had active viral infections (see below).

### 3.6. Viral Sequences

Next generation sequencing approaches such as RNA-seq seem ideal methodologies to delineate and to sample the plant-pathogenic virome of plant feeding insect vectors such as squash bugs. Aphids and whiteflies are the major vectors of cucurbit viruses. Squash bugs are known vectors of *Serratia marcescens*, the bacterial pathogen of Cucurbit yellow vine disease [42,60,61]. The newly founded laboratory squash bug colony from which RNA pools used in this study were isolated was comprised of animals recently collected from local squash and zucchini fields. The animals also were occasionally fed with squash and zucchini cuttings brought in from the field or purchased at local organic outlets. Thus it was not entirely unexpected that RNA-seq revealed tags homologous to many RNA and DNA viruses known to infect insects, or to be vectored to plants by insect species.

To obtain a preliminary survey of the *A. tristis* virome all reads from the combined RNA pools that did not align to the Trinity assembly were BLASTx screened against the NCBI gbvrl database resulting in 915 significant hits logged. Major taxa of insect viruses detected within these hits included ascoviridae, iridoviridae, granuloviridae, entomopoxviridae, baculoviridae, nudiviridae, even ichnoviridae and bracoviridae. Among these were sequences of plant infecting viruses sequences were detected of viroids, bromoviridae, closteroviridae, genimiviridae, luteoviridae, potyviridae, secoviridae, tombusviridae and virgaviridae. The presence of several of the 17 cucurbit associated viruses such as Cucurbit aphid-borne yellows virus, Melon yellow spot virus, and Papaya ringspot virus within the squash bug colony was indicated [62]. Transcripts from DNA viruses also would have been sampled by this approach and thus even RNA/DNA viruses of vertebrates also were observed. These findings set the stage for a more comprehensive survey of plant pathogens harbored by squash bugs in the laboratory and the field. Confirmation that these viruses occur within wild populations of squash bugs would indicate that further prospecting for entomopathogenic viruses effective as biological control agents of *A. tristis* is warranted. Next generation deep sequencing of small RNAs extracted from infected cells or insects yields short interfering RNAs, which are products of the Dicer-dependent antiviral pathway, could enable the *in silico* reconstruction of viral transcripts or entire RNA/DNA virus genomes [63,64,65,66,67]. Phloem/xylem feeding insects can also be used to sample the viral diversity of plant populations on which they are feeding (“vector-enabled metagenomics”) [68,69]. Viromes of insects [67,70,71], their food plants [72,73], their predators [74], and even their entomopathogens could be sampled simultaneously *in toto*. Transcriptomes and genomes have recently been completed for several cucurbits [75] which would allow concurrent pathogenomic monitoring of squash bug, virus and cucurbit host transcription during insect feeding, e.g., [76]. 

**Table 4:** Expression profiling of *Anasa tristis* RNA interference pathway genes in response to bacterial or fungal elicitation 18 h post inoculation.

Transcript	NR Annotation	Mean Control ^a^	Mean Bacterial ^a^	B/C ^b^	Mean Fungal ^a^	F/C ^b^	NRid	Score	Eval	Best Match
10966c0seq13	argonaute-1	33	34.96	1.06	43	1.3	gb|ACO40482.1	133	5E-29	*Nasonia vitripennis*
1860c0seq1	argonaute-2	1233.65	1107.58	0.9	634.29	0.51	ref|XP001607164.1	721	0	*Nasonia vitripennis*
108824c0seq1	aubergine	0.67	0	0	0	0	ref|NP001159378.1	221	2E-57	*Apis mellifera*
44766c0seq1	dicer-1	9	14	1.56	11.67	1.3	emb|CAX68236.1	754	0	*Blattella germanica*
12963c0seq2	dicer-2	11.08	1.69	0.15	12.46	1.12	ref|NP001107840.1	793	0	*Tribolium castaneum*
12963c0seq3	Endoribonuclease Dcr-1	94.37	110.64	1.17	75.62	0.8	gb|EFN62420.1	432	1E-120	*Camponotus floridanus*
17917c0seq1	microprocessor subunit DGCR8	91.33	130.33	1.43	69	0.76	ref|XP003397039.1	575	1E-162	*Bombus terrestris*
7746c0seq1	PIWI	235.33	304.67	1.29	125	0.53	ref|XP001652945.1	722	0	*Aedes aegypti*
12734c0seq1	RISC-loading complex subunit	92.92	51.74	0.56	58.93	0.63	ref|XP001601132.2	380	1E-104	*Nasonia vitripennis*
2817c0seq1	Translin-like	327.0	373.67	1.14	213.0	0.65	dbj|BAG65665.1	611	1E-174	*Nasonia vitripennis*

^a^ Average number of reads/contig (three biological replicates); ^b^ Ratio of reads, Bacterial/Control, or Fungal/Control.

## 4. Conclusions

To construct a reasonably complete *A. tristis* immunotranscriptome, adults were subjected to microbial elicitation of the immune response. The resulting *de novo* assembly contained many *A. tristis* orthologs of immune system proteins known from other insect species, including those of phylogenetically related hemipterans. Key components of the entomopathogen recognition system, humoral and cellular immune responses, and second messenger regulatory networks were identified. Interestingly, a partial virome of *A. tristis* was noted within the *de novo* assembly, along with the presence of known insect transmitted bacterial species which will become the subject of future studies. Novel approaches are needed to target the unique mode of *A. tristis* feeding on the phloem of its cucurbit hosts. The *de novo* transcriptome generated in this report consists of the vast majority of transcript categories synthesized by this insect to support life processes, such as olfaction, neuroendocrinology, reproduction, digestion, and the immune response against bacterial and fungal elicitation. Targeted disruption of one or more of these transcripts, or the proteins that they encode, could reduce the severe economic impact of *A. tristis* on horticultural production.

## Disclaimer

The U.S. Department of Agriculture (USDA) prohibits discrimination in all of its programs and activities on the basis of race, color, national origin, age, disability, and where applicable, sex, marital status, familial status, parental status, religion, sexual orientation, genetic information, political beliefs, reprisal, or because all or part of an individual’s income is derived from any public assistance program. (Not all prohibited bases apply to all programs.) Persons with disabilities who require alternative means for communication of program information (Braille, large print, audiotape, *etc*.) should contact USDA’s TARGET Center at (202) 720-2600 (voice and TDD). To file a complaint of discrimination, write to USDA, Director, Office of Civil Rights, 1400 Independence Avenue, S.W., Washington, D.C. 20250-9410, or call (800) 795-3272 (voice) or (202) 720-6382 (TDD). USDA is an equal opportunity provider and employer.
